# High-Risk Multiple Myeloma: Integrated Clinical and Omics Approach Dissects the Neoplastic Clone and the Tumor Microenvironment

**DOI:** 10.3390/jcm8070997

**Published:** 2019-07-09

**Authors:** Antonio Giovanni Solimando, Matteo Claudio Da Vià, Sebastiano Cicco, Patrizia Leone, Giuseppe Di Lernia, Donato Giannico, Vanessa Desantis, Maria Antonia Frassanito, Arcangelo Morizio, Julia Delgado Tascon, Assunta Melaccio, Ilaria Saltarella, Giuseppe Ranieri, Roberto Ria, Leo Rasche, K. Martin Kortüm, Andreas Beilhack, Vito Racanelli, Angelo Vacca, Hermann Einsele

**Affiliations:** 1 Department of Internal Medicine II, University Hospital, 97080 Würzburg, Germany; 2 Department of Biomedical Sciences and Human Oncology, Section of Internal Medicine “G. Baccelli”, University of Bari Medical School, 70124 Bari, Italy; 3 Orthopedics and Traumatology Unit ASL BA-Ospedale della Murgia “Fabio Perinei”, 70022 Altamura, Italy

**Keywords:** multiple myeloma, angiogenesis, extramedullary disease, drug resistance, bone marrow microenvironment

## Abstract

Multiple myeloma (MM) is a genetically heterogeneous disease that includes a subgroup of 10–15% of patients facing dismal survival despite the most intensive treatment. Despite improvements in biological knowledge, MM is still an incurable neoplasia, and therapeutic options able to overcome the relapsing/refractory behavior represent an unmet clinical need. The aim of this review is to provide an integrated clinical and biological overview of high-risk MM, discussing novel therapeutic perspectives, targeting the neoplastic clone and its microenvironment. The dissection of the molecular determinants of the aggressive phenotypes and drug-resistance can foster a better tailored clinical management of the high-risk profile and therapy-refractoriness. Among the current clinical difficulties in MM, patients’ management by manipulating the tumor niche represents a major challenge. The angiogenesis and the stromal infiltrate constitute pivotal mechanisms of a mutual collaboration between MM and the non-tumoral counterpart. Immuno-modulatory and anti-angiogenic therapy hold great efficacy, but variable and unpredictable responses in high-risk MM. The comprehensive understanding of the genetic heterogeneity and MM high-risk ecosystem enforce a systematic bench-to-bedside approach. Here, we provide a broad outlook of novel druggable targets. We also summarize the existing multi-omics-based risk profiling tools, in order to better select candidates for dual immune/vasculogenesis targeting.

## 1. Introduction

One of the first attempts to stratify multiple myeloma (MM) patients is based on the commonly-available parameters that may predict the risk factor profile, identify different treatment response [1], and quantify tumor cell burden. This classification was known as the Durie–Salmon (D&S) clinical staging. However, it does not account for biologic disease variability, and it is affected by observer-related bias in the quantification of lytic lesions on the skeletal survey [2]. Moreover, the clinical practice indicates that progression-free survival (PFS) is strongly correlated to the success of autologous stem cell transplantation (ASCT) [3]. Since 2005, the D&S has been replaced by the International Staging System (ISS), which is a prognostic model based on β2-microglobulin and albumin [4]. The identification of these two parameters allows stratification into three classes of risk, impacting overall survival (OS). Although this system is simple and reproducible, it does not take into account the cytogenetic alterations that entail another fundamental prognostic factor and neglect the MM milieu’s role in tumor aggressiveness [3,5]. The genetic event’s role in MM pathogenesis has been described as a multistep process, affecting the neoplastic clone through the primary and secondary aberrations’ acquisition, which unmistakably contribute to the progressive acquisition of an aggressive phenotype. The MM microenvironment actively supports the MM disease evolution, also impacting the drug-resistant disease behavior. 

Here, we review the available evidence in order to formulate a comprehensive risk-driven patient approach.

## 2. Genetic Determinants of Multiple Myeloma and Clinical Prognostic Scores

### 2.1. Primary and Secondary Genetic Events

**Figure 1:**
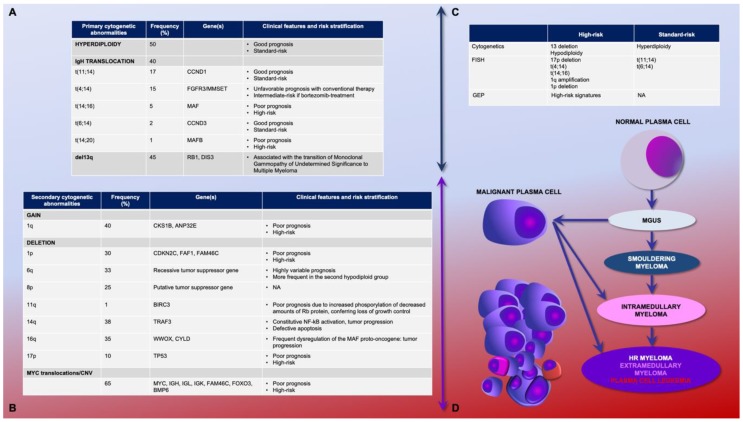
Relationship between peculiar cytogenetic abnormalities and multiple myeloma evolution. (**A**) Primary genetic events occur in the early premalignant phase during the transition from a normal plasma cell to a clonal plasma cell; (**B**) secondary genetic events occurring during the disease progression [6]; (**C**) genetic risk stratification, modified from [5]; (**D**) multiple myeloma disease evolution. GEP: gene expression profile. MGUS: monoclonal gammopathy of undetermined significance.

Primary genetic events leading to clonal proliferation are represented by hyperdiploidy, chromosomal structural abnormalities, and recurrent translocations. In 20% of MM patients, the juxtaposition of the immunoglobulin enhancer with the coding gene regions nearby, oncogenes, results in their constitutional overexpression [3,5,6] (Figure 1A). The secondary genetic events are mainly numerical alterations, such as deletion, gain of chromosomes, and specific gene expression alterations and mutations, for instance c-myc and RAS, respectively [3,5,6,7,8] (Figure 1B). In MM, the most common cytogenetic finding is hyperdiploidy, found in 50% of patients; usually, it implies a good prognosis with an OS of 7–10 years, namely considered as a standard risk. Nevertheless, the underlying biological mechanisms are still unknown, as well as the prognostic role of distinctive karyotype gains [9,10].

Importantly, the group of translocations that involves chromosome 14 globally accounts for 40% of patients and includes good-prognosis forms such as *t*(11;14). However, the *Stichting Hemato-Oncologie voor Volwassenen Nederland* (HOVON—EudraCT no. 2004-000944-26) trial using bortezomib in induction prior to high-dose melphalan therapy and bortezomib maintenance, overcame the increased risk of *t*(4;14) considering it as a standard risk if bortezomib-containing therapies are applied [11,12,13,14]. Rare translocations may also occur; *t*(14;16) indicates an aggressive phenotype, and it is associated with high free light chain level and acute renal failure (25% of patients); *t*(14;20) is characterized by an aggressive disease per se [6,7,8]. MM high-risk features include alterations of chromosome 17 and chromosome 1. The 17p deletion at diagnosis occurs in about 10% of patients, and it is frequently acquired after therapy; 40% of patients harbor 1q amplification, often associated with IgH translocations or with 1p deletion [15] (Figure 1C). The disease evolution follows the abovementioned pathogenetic events (Figure 1D).

Combined lesions, besides the type of cytogenetic anomalies, define the MM prognosis. In 1069 newly-diagnosed MM (NDMM) enrolled in the *Medical Research Council* (MRC IX—ISRCTN68454111) trial, a strong positive association with IGH and 1q gain was found: 72% of IGH translocations were harboring 1q gain and 12% del(17p), and 4% showed all three unfavorable markers. Indeed, genetic abnormalities are not isolated events since they can occur together, conferring an additive effect on OS [15].

### 2.2. Genetic Prognostic Relevance: Gene Expression Profiling and Cytogenetics

Gene expression profiling (GEP) represents an additional tool to assess the MM genetic heterogeneity [16,17]. A 70-gene microarray panel may characterize molecular MM subgroups and signatures associated with high-risk diseases and short survival. This approach identified prognostic relevant molecular determinants on chromosome 1: the upregulated genes were mapped on 1q and the downregulated ones on 1p. The high-risk score obtained from the expression levels predicted a shorter duration of disease remission, event-free survival, and OS [18]. Moreover, del17, 1q gain, and *t*(4; 14) detected by fluorescence in situ hybridization [16] are strongly associated with a 16–24% high risk, regardless of treatment, age, and disease status [19]. Recently, an International Myeloma Working Group (IMWG) consensus defined NDMM patients harboring unfavorable cytogenetics and GEP adverse molecular signatures as high risk (Figure 1C) [5].

### 2.3. Combined Scores and Clinical Predictors of Prognosis

Combined scores have been extensively validated [20,21]. Accordingly, a Revised International Staging System (R-ISS) has been developed. A total of 3060 NDMM patients were enrolled into 11 international, multicenter clinical trials. All patients received either immuno-modulatory agents (IMiDs) or proteasome inhibitors (PIs) [22]. The R-ISS was able to identify three populations with different outcomes in terms of relapse incidence and OS [22]. By using univariate analysis, the R-ISS III turned out to be the stage with the highest prognostic impact compared to the remaining individual parameters in terms of both PFS and OS. Bolli et al. reported a large number of sequencing data from a 418 NDMM cohort. Gene mutational status joined with copy number aberrations and translocations led to the identification of patient subgroups with different outcomes. Notably, chromosome 6 deletion, involving PRDM1 combined with *t*(4;14) or BIRC2/3 deletion, identified individuals with worse OS. Moreover, four different clusters were identified based on genetic compositions with different PFS and OS. The worst prognostic features were associated with cluster 2, including 1q amplification, a higher IGH translocation and TP53 mutations rate, and deletions of 17p, 13q, BIRC 2/3, and XBP1 [23]. Walker et al., in a comprehensive genomic analysis performed on more than 800 patients, described and validated a peculiar MM population characterized by poor prognosis; the double-hit MM are defined as diseases harboring a bi-allelic inactivation of TP53 or an amplification of chromosome 1 involving the CSK1B gene together with ISS3. The poor prognosis associated with the population characterized by these alterations makes the recognition of this genetic subset advisable [24].

The clear distinction between low-, intermediate-, and high-risk groups was also confirmed by diversifying the analysis for different types of therapy, i.e., whether or not a high-dose regimen supported by ASCT was employed or whether IMiDs-based versus PIs-based treatments were given [22]. Another fundamental prognostic indicator is the depth of response [25,26,27]. Indeed, the complete remission (CR) achievement was associated with a significant increase in OS in NDMM regardless of transplant eligibility; this was also confirmed in the relapsed/refractory group (RRMM) [25,26,27,28]. 

The depth of response takes on a particularly critical value when the patients are stratified according to the cytogenetic risk [29]. Undeniably, failing to obtain a CR in high-risk patients, as defined by the GEP signature, correlates with a significant reduction in OS [30]. In addition, the association between the persistence of post-transplant residual disease identified by flow cytometric immune-phenotyping (fluorescence-activated cell-sorting (FACS)) and the presence of a high-risk basal cytogenetic profile is characterized by unfavorable outcomes [31].

### 2.4. Minimal Residual Disease

Disease remission is commonly defined by serological and immunological parameters [32]; these are not sensitive enough to detect the smallest residual tumor burden [33,34]. 

Therefore, over the past few years, the response assessment paradigm has been integrated with more precise tools able to detect minimal populations of clonotypic plasma cells (PCs) in the bone marrow (BM) [35]. 

As in other hematological malignancies [36], the minimal residual disease (MRD) status in MM, defined as the clonotypic PCs’ persistence in the BM after therapy, is emerging as an ultra-sensitive tool, showing a deep impact on survival. In particular, two main methods have been validated for the detection of MRD based on next-generation FACS (NGF) and next-generation sequencing (NGS) [37].

Martinez Lopez et al. designed an NGS-based method where the PCs are bar-coded by their clonotypic immunoglobulin rearrangements, accurately identifying the neoplastic clone. This method is superior to the one based on standard eight-color FACS. MRD-negative patients showed significantly higher survival [33,38,39]. Furthermore, the NGF is turning out to be an ultra-sensitive tool for MRD detection. Flores-Montero et al. analyzed a 63-patient cohort with a new MRD panel for FACS, showing that NGF-MRD is superior to the standard eight-color FACS. NGF was able to identify residual sub-clones that had barely been detected by other methods [40]. The MRD negativity confirmed its impact on the clinical outcome [41]. Nonetheless, the MRD standardization and the real impact on the patient’s management remains an unmet clinical need. Both techniques bring advantages and disadvantages that raise a non-negligible challenge in selecting the best option. Both of them are characterized by a broad applicability along different laboratories, and both have significantly increased their sensitivity, being able to detect at least one cell for every 100,000. In order to reach a high sensitivity, NGF needs to acquire and analyze five millions of events in comparison to the NGS method, which would reach the same level of depth with less than one million cells [35]. Conversely, NGF appears faster and more reproducible, relying on fresh sample processing and automated flowchart analysis. NGS invariably depends on baseline sample availability, is time consuming, and implies bioinformatic-based analysis. The two described methods would depict the residual disease only from a single biopsy in a single specific body region, potentially missing the typical MM spatial distribution and heterogeneity [35,42]. Moreover, the assessment can be biased at several layers, such as the aspiration volume and peripheral blood dilution.

Thus, the imaging techniques are acquiring a central role in the initial work-up and in the response assessment [37,43,44,45].

The magnetic resonance and low-dose radiation computer tomography scans are now considered the gold standard for the initial NDMM assessment. IMWG defined the guidelines for the positron emission tomography (PET)-guided scan in MM. The Positron emission tomography with 2-deoxy-2-(fluorine-18)fluoro- D-glucose integrated with computed tomography (18F-FDG PET) scan represents the most common tool for detection of active metabolic MM lesions, although the technique may be hindered by a lack of sensitivity and specificity [44]. Metallic implants might lead to false positive results, as well as inflammatory states; alternatively, the patients’ hyperglycemia and steroid therapy that transiently suppress the metabolic state can enhance the false-negative rate [43]. Rasche et al. found that hexokinase-2-low expression can also reduce the diagnostic sensitivity, due to the FDG phosphorylation decrease and subsequent lower uptake by tumor cells [46,47].

In order to increase the accuracy of PET diagnosis, alternative metabolic pathways have been proposed as new targets [48]. Lapa et al. evaluated the usefulness of the radiotracers 11C-methionine (MET) and reported a potential diagnostic superiority of MET-PET/CT in comparison to FDG for staging and re-staging of both intra- and extra-medullary MM lesions. MET uptake correlated with BM involvement and seemed to be a more accurate marker of tumor burden and disease activity compared with the standard 18F-FDG PET [48]. On top of this, the possible use of the chemokine (C-X-C motif) receptor (CXCR4) holds promise to be a target-tracer for MM imaging and endo-radiotherapy. CXCR4 represents an attracting molecule that could at the same time be able to inform about the tumor infiltration and its immune-environmental counterpart [49] and could select patients suitable to CXCR4-directed therapies. Lapa et al. reported successful, but transient remissions in heavily-pretreated patients with relapse/refractory MM and extramedullary disease who underwent CXCR4-directed endo-radiotherapy, demonstrating that this treatment is feasible and successful even at an advanced MM stage [50]. 

In the near future, radioligand therapy along with imaging technology can significantly improve the diagnostics and MRD assessment. 

## 3. Aggressive and Refractory Multiple Myeloma Phenotypes: The Neoplastic Clone and the Interaction with the Tumor Microenvironment

### 3.1. The Angiogenic Trigger in Multiple Myeloma: Novel Perspectives from the Immune Microenvironment

MM is considered, from a geno-/pheno-type point of view, halfway between a solid and a hematological neoplasia. A potential explanation is provided by the huge impact of the tumor associated immune-microenvironment and its angiogenic potential, which plays a major role in the disease pathogenesis and progression [51]. Of note, GEP70 includes in its 70 high prognostics genes markers related to angiogenesis and to the control of tumor-immune response. This panel comprises genes such as FABP5 [52], BIRC5 [53], AURKA [54], ALDOA [55], YWHAZ [56], and ENO-1 [55], strong mediators of neo-vasculogenesis. Recently, Saltarella et al. published the results of the *Gruppo Italiano Malattie Ematologiche dell’Adulto* (GIMEMA-MM0305 NCT01063179) clinical trial, where patients were randomized between two different therapy schedules (bortezomib-melphalan-prednisone-thalidomide followed by bortezomib-thalidomide maintenance vs. bortezomib-melphalan-prednisone); the enrolled subjects were also studied for several serum angiogenic factors at different time points. The authors concluded that high levels of VEGF and FGF-2 were associated with a bad prognosis [57].

Thus, enhanced angiogenesis strongly impacts MM prognosis due direct and indirect triggers of MM-cell survival [58]. The cytokine- and cell-adhesion-dependent BM milieu support new vessel formation and MM proliferation, irrespective of immune-surveillance. Leone et al. provided evidence that the intimate interaction between ECs, MM, and CD8+ T cells creates a permissive immune-microenvironment within BM that allows undisturbed MM proliferation. They demonstrated that ECs act as antigen-presenting cells, stimulating a central memory CD8+ T cell population, which negatively regulates the effector memory CD8+ T cells with anti-tumor activity. Remarkably, a defective immunosurveillance allows for the persistence and proliferation of MM cells: an immune-microenvironment disease evolution characterized by exhausted CD8+ cells, overexpressing check point molecules such as LAG3 and PD1, in preclinical models offers suitable targets for increased survival in in vivo models [59]. In a clinical setting, a patient with a larger CD8 cytokine profile, along with competent CD8 T cells and dendritic cells had an increased OS and time to progression [60]. Therefore, it is likely that new blood vessel formation (i.e., angiogenesis) within BM, a recognized hallmark of MM progression, parallels MM evasion from T cell immune surveillance [61,62,63]. Moschetta et al., highlighted how endothelial-progenitor-cell trafficking is implicated in MM progression, especially in the early disease phases [64]. Several clinical trials in MM tested the effects of bevacizumab used in combination with other agents, including lenalidomide, dexamethasone, or bortezomib with discouraging results [65]. In addition to bevacizumab, other VEGFRs targeting compounds (including aflibercept-VEGF-trap), activated pathway inhibitors (tyrosine kinase, PI3K/Akt-MEK/ERK, FAK), anti-cytokine drugs, and monoclonal antibodies have shown an anti-angiogenic effect, but not sufficiently to enter in the clinical MM setting [65,66,67,68,69,70,71,72,73]. Therefore, this evidence provides the translational rationale to overcome the scanty effect of the anti-angiogenic approach in MM obtained so far [74]. Assuming the different angiogenic impacts on a given disease stage, it would be worth better tailoring the vasculogenic manipulation in the early MM with the high-risk phenotype [64,75]. In this frame of thinking, one critical effect of corrupted angiogenesis is disease dissemination, within and outside the bone marrow, driving intra- and extra-medullary MM manifestation [76].

### 3.2. Extramedullary Disease Characterization as a Paradigm for Corrupted Interaction between MM cells and Its Ecological Niche

Based on the acquired molecular advantages and the prone immune-microenvironment, MM cells are able to follow chemotactic signals and to colonize different BM compartments [76], especially in the later phases of the disease [43].

Extramedullary disease (EMD) has been considered as the organs’ colonization other than bone by infiltrating PCs [77]. Among these conditions, plasma cell leukemia represents a rare, but aggressive phenotype of extramedullary dissemination where PCs lose their “homing” capacity to the BM compartment completely [78,79]. 

The incidence is 6–8% in NDMM and rises to 10–30% in RRMM [79,80,81]. The sites mostly involved are liver, skin/soft tissue, pleural effusion, kidneys, lymph nodes, pancreas [82], and the central nervous system (CNS), hence representing a challenge for clinical practice [83,84,85]. The common biologic characteristics are: higher LDH level, anemia, thrombocytopenia, non-secretory MM, high-risk GEP and cytogenetics, and immature/plasmablastic morphology [80,86]. The clinical approach comprises physical examination with CNS assessment and functional whole-body imaging [85]. The EMD presence at disease onset is associated with poor PFS [80,81], and it results in an even more aggressive behavior when it affects directly soft tissues not anatomically related to the BM [87,88]. 

Regarding plasma cell leukemia (PCL), it is diagnosed when more than 20% of PCs are detected in the peripheral blood (absolute PC count above 2 × 10^9^/L). It is frequently associated with leukopenia due to dysplastic BM or heavy previous significant treatment exposure [82]. It occurs in 2–4% of MM patients, and it is classified as primary or secondary. Its primary form (60–70% of cases) arises in absence of a pre-existing MM; the secondary one (30–40% of cases) represents an end-stage MM leukemic transformation [41]. The prognosis is very poor with an OS rate remaining below 10% during five years in the primary PCL [41] and only one month in secondary PCL [82]. Indeed, PCLs are characterized by abnormal immunophenotype and high-risk cytogenetics (most frequent: hypodiploidy, *t*(11;14), 1q gain, and del17p) [78,82].

From a biological point of view, MM dissemination out of the BM is related to the expression of adhesion molecules and chemokine receptors [76,80,81]. EMD is characterized by BM microenvironment-independent tumor growth, inhibition of apoptosis, escape from immune surveillance, and drug resistance (DR), which pinpoint this condition as a high-risk feature [79]. 

**Figure 2:**
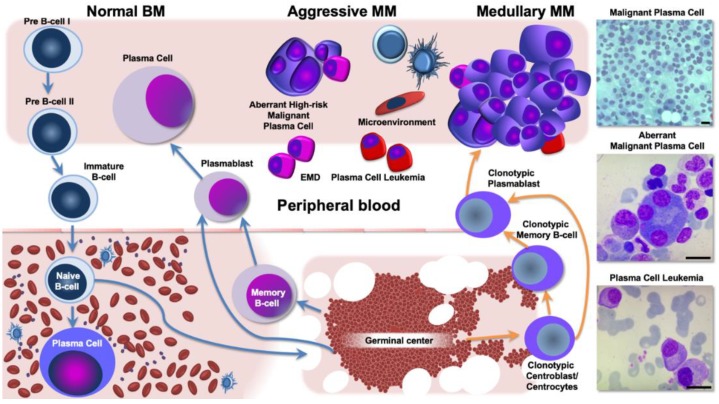
B-cell differentiation, multiple myeloma (MM) development, and aggressive disease phenotypes. Left panel: pre-B cells migrate from the bone marrow (BM) into the peripheral blood and the germinal center. Memory B-cell differentiation drives the production and localization of plasma cells (PCs) into the BM. Right panel: The earliest clonotypic cell, putatively the MM precursor, can turn into mature premalignant PCs, namely MGUS. Subsequent genetic events lead to overt disease in multiple BM sites. Ultimately, clonal evolution driven by disease biology and BM microenvironment interaction continues to select MM PCs that finally give rise to extramedullary and aberrantly growing sub-clones. EMD: extramedullary disease.

Extrinsic and intrinsic factors are involved in the MM extramedullary localization. Tumor heterogeneity, concerning the acquisition of genetic lesions able to modify the malignant plasma cells’ interaction with their microenvironment, is mainly responsible for the spreading of MM. In more detail, acquisition of BRAF or other activating RAS pathway mutations reduced expression of adhesion molecules or chemokines, altered SDF1/CXCR4 axis interaction, and enhanced angiogenesis as drivers of MM disseminations (Figure 2) [80,88,89,90,91,92]; the MM niche represents an environment where the tumor is able to proliferate, taking advantage of a protective milieu composed by activated stromal and endothelial cells, capable of promoting invasion and angiogenesis. An exhausted immune compartment facilitates MM progression and sustains a permissive soil [75].

Moreover, about 30% of patients with EMD at diagnosis are considered high-risk due to poor first-line therapy response and genetic characteristics [81]; these patients could suffer a primary refractoriness status or early (within one year) relapse occurring after the therapeutic intervention or during the maintenance protocol [81,93,94]. Therefore, a deeper understanding of the molecular basis that enables the rise of this unfavorable phenotype is mandatory in order to provide a more efficient treatment for these selected patients [95].

### 3.3. Biological Background and Genomic Landscape of High-Risk Multiple Myeloma

The spatial genetic heterogeneity determines differential proliferation potential within the BM or in extra-medullary sites, depending on different clones and sub-clones with a variety of genome alterations [96]. 

Given spatial differences, commonly-used prognostic markers are del(17p) in 33% of patients and translocations involving MYC in 25%. The 1p deletion and 1q21 gain/amplification are frequently shared between different spatial sites, with 19% of patients presenting a regionally-restricted event [96]. Loss of heterozygosity, involving 1q, present in 21% of patients, as well as changes in chromosomes 1, 4, 5, and 8 are the most frequent contributors to spatial heterogeneity. Moreover, the most recurrent mutated genes are NRAS, KRAS, TTN, ROBO2, TP53, and BRAF. On the contrary, gene alterations involved in the mitogen-activated protein kinase (MAPK) pathway are the most important mutations concurring with site differences [96,97,98,99]. To summarize, the spatial heterogeneity harbors a molecular signature that often characterizes advanced disease stages. Of note, the serine-threonine kinase BRAF has been found to be mutated in 5–10% [100,101] of all MM patients, and the BRAFV600E mutation is one of the most common variants [77,96,102]. Targeting BRAFV600E has been employed in several neoplastic disorders with clinical benefit [103,104]. Gaining this mutation in MM was linked to increased EMD incidence, shortened PFS, and reduced OS [89]. Therefore, Raab et al. used vemurafenib, a BRAFV600E-specific inhibitor, to treat resistant EMD harboring this mutation, obtaining a variable grade of disease control [89,105]. Nonetheless, when NRAS mutations were acquired determining vemurafenib resistance, bortezomib showed clinical efficacy on resistant clones, conferring a good disease control [105]. 

Besides driver cell genome alterations, different mutations have been described in different sites as a non-sequential model in MM. This evolutionary selective pressure could explain the selection of decreased BM-dependent clones, able to grow within the EMD sites (Figure 2) [96,102,106].

## 4. Mechanisms of Drug Resistance in Aggressive Multiple Myeloma

Despite the direct targeting of oncogenomic drivers and the availability of new compounds that improved MM treatment, the therapeutic pressure can also select resistant mutated neoplastic clones [107,108]. In MM many ways by which the disease develops drug resistance (DR) have been identified; genomic instability and tumor microenvironment are two of the main triggers of DR and clonal evolution [78].

### 4.1. New Insights from the Bone Marrow Microenvironment Adhesion-Mediated Drug Resistance

**Figure 3:**
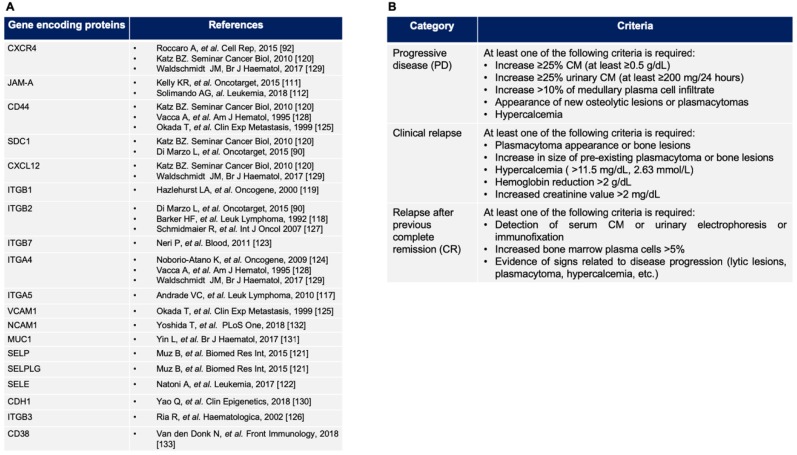
(**A**) Gene encoding protein list: adhesion molecule implicated in drug resistance described in MM (see the text and [117,118,119,120,121,122,123,124,125,126,127,128,129,130,131,132,133] for details); (**B**) definition of the disease relapse according to the International Myeloma Working Group (see the text for details). M protein: monoclonal protein.

The BM niche’s pivotal role in DR acquisition derives from several factors [109]; one of the main refractoriness drivers is the adhesive interaction between PCs and BM stromal cells and extracellular matrix components [90]. Moreover, cell adhesion triggers the epithelial to mesenchymal transition (EMT) and metastatic process in solid tumors [110]. Roccaro et al. investigated the function of CXCR4 and found this molecule as an EMT regulator in MM. PCs over-expressing CXCR4 are more prone to bone dissemination when transplanted to an in vivo model (Figure 3A). On the contrary, CXCR4-silenced PCs resulted in both reduced bone homing and cell growth. Furthermore, ulocuplumab, an anti-CXCR4 monoclonal antibody (mAb), modifies the RNA expression of signals that mediate EMT, reducing tumor size and tumor BM homing [92].

Another cell adhesion molecule (CAM) that plays a major role in MM survival is the junctional adhesion molecule-A (JAM-A) [111,112]. It resulted in lower expression on PCs derived from MGUS than in MM patients; remarkably, among MM patients, different JAM-A surface level (JAM-A^high^ versus JAM-A^low^) implied worse PFS in the JAM-A^high^ group [111,112]. Moreover, soluble JAM-A levels displayed a direct correlation to bone lesion in newly-diagnosed patients, as well as to PCs’ infiltration at disease relapse [112]. The JAM-A silencing resulted in reduced MM cell migration and colony formation [112]. Similar results were found in MM in vivo models treated with an anti-JAM-A mAb [112]. Overlapping findings derived from studies on CD44, known as a β-catenin transcriptional target, which is a functional component of the CAM, and it is another potential mediator of DR [113]. CD44 is overexpressed on PCs derived from IMiDs-resistant patients and mediates lenalidomide resistance. As in other hematologic neoplasia, blockade of adhesion molecules and their downstream pathways [114,115], such as CD44 either with mAb, gene-silencing or all-trans-retinoic acid, reduced adhesion and restored drug sensitivity (Figure 3A) [113,116].

### 4.2. Immuno-Modulatory Agents 

One of the paramount drugs employed in MM is lenalidomide. Though very effective, MM patients can develop primary or secondary resistance to it. It has been found that lenalidomide binds CRBN, which participates in the constitution of the E3 ubiquitin ligase (CRL4) complex [134]. Lenalidomide also reduces two transcription factors, Ikaros (IKZF1) and Aiolos (IKZF3). Mutations involving CRBN and IKZF1/3 binding sites confer resistance to IMiDs and are clinically significant [108,134,135]. This mutational status assessment could provide useful tools to drive clinical decisions.

The Multiple Myeloma German Study Group (DSMM) has discovered that in standard-risk patients, adverse PFS and OS have been associated with high expression levels of IKZF1 and IKZF3 [136]. Nonetheless, Zhu et al. in heavily-pretreated patients showed that low levels of Ikaros and high levels of KPNA2 were associated with poor prognosis in univariate analysis [137]. Basserman et al. have recently described an alternative IMiDs mechanism of action involving the CD147-MCT1 complex. This machinery is involved in cellular proliferation and survival and is able to induce invasion and angiogenesis by a direct regulation of metalloproteinase expression or the vascular endothelial growth factor. Moreover, an overexpression of the CD147-MCT1 complex is correlated to Lenalidomide resistance both in in vitro and in vivo models [138]. Moreover, also the epigenetic regulators such as EZH2 could mediate IMiD drug resistance and patients, with this poor prognosis signature could benefit from epigenetic modifier-targeted therapies [139].

### 4.3. Proteasome Inhibitors 

Other milestones in MM treatments are PIs [140]. In vitro, continuous exposure to bortezomib and analogues generates resistant cell lines. Single-point mutations in PSMB5 have been described as the underlying cause of this resistance because of a conformational or steric change to the proteasome drug-binding site, reducing PIs’ pharmacological interaction [141]. This mutation is usually absent at diagnosis. PSMB5 acquires new mutations in less than 5% of patients after multiple PI treatments, thus conferring DR [142].

Mitra et al. [143] analyzed the drug response of individual cells based on target transcriptome in pretreatment cell analysis, thus predicting PIs-resistance, i.e., the residual resistance affected the PI treatment response [143]. Another PI resistance mechanism is determined by the downregulation of the proteasome 19s subunit due to an impairment of ATPase activity [144]. Taken together, the downregulation of proteasome subunits and the acquisition of mutation affecting the drug’s mechanism of action could explain at least 10% of the acquired resistance in MM patients.

In addition, the downregulation in the tight junction and the proangiogenic genes resulted in PI resistance. TJP1 [145] and HGF/c-MET [146,147] have been identified as determinants of PIs’ susceptibility. Indeed, TJP1 knockdown preserved cell viability after the exposure to PIs, also decreasing apoptosis, and conferring resistance in the presence of wild type or mutant RAS. On the contrary, TJP1 overexpression sensitized MM cells to PIs [145]. Zhang et al. demonstrated that TJP1 suppressed EGFR/JAK1/STAT3 signaling, thus having great clinical relevance in terms of PFS and response to therapy [145]. Remarkably, the HGF/cMET loop sustains DR [147] and angiogenesis [146] and represents an attractive tool that targets the neoplastic clone and the microenvironment, potentially overcoming therapy resistance [148].

Interestingly, in several in vitro and in vivo systems, both MM and stromal cells, such as fibroblasts [149,150], osteoclasts [151], and endothelial cells [152], recruited in the tumor milieu seem to stimulate the proliferation and to drive the immune permissive microenvironment [62], thus representing a new attractive therapeutic target.

## 5. Approach to the Patient with High Risk Related to Relapsed/Refractory Multiple Myeloma 

A refractory MM is defined by an insensitivity to three or more courses of anti-myeloma therapy or that has progressed within 60 days of the last treatment. Primary refractory MM patients are the ones that never experienced a partial response to all previous lines of therapy; the relapsed patients are the ones who required a new rescue therapy after a partial or complete remission interval of at least 60 days. The definition of the disease relapse follows the criteria of the International Myeloma Working Group (IMWG) (Figure 3B) [153].

### 5.1. Validated Therapy for Relapsed/Refractory Multiple Myeloma

The duration, the quality, and the depth of response to previous therapy represent fundamental principles to take into account for the choice of the relapse/refractory treatment program. Moreover, a complete RRMM framework needs to consider high relapse risk clinical features (systemic symptoms, organ damage, EMD, circulating plasma cells increase LDH), acquired high-risk FISH cytogenetics lesions (17p deletion, chromosome 14 translocations, alterations involving chromosome 1), and residual therapy-related toxicity derived from previous treatments [154]. 

**Figure 4:**
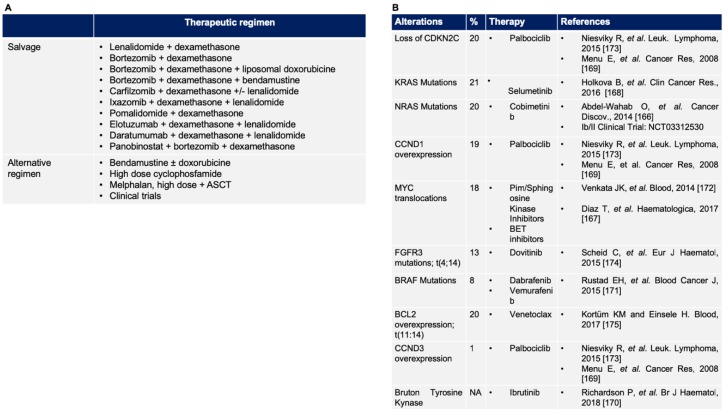
(**A**) Therapeutic regimens for relapse/refractory MM; (**B**) common druggable molecular alterations in MM (see the text and [166,167,168,169,170,171,172,173,174,175] for details). ASCT, autologous stem cell transplantation.

Anti-angiogenic drugs such as lenalidomide and pomalidomide represent the back-bone of the treatment schedules; in particular, lenalidomide was firstly approved in combination with bortezomib and dexamethasone [155] in 2015 and one year later with the second-generation proteasome inhibitor carfilzomib (Figure 4A) [156].

Bortezomib in combination with dexamethasone (bortezomib-dexamethasone—VD) [157] or the triple-therapy with also liposomal doxorubicin (bortezomib, doxorubicin and dexamethasone—PAD) [157] and lenalidomide-dexamethasone (lenalidomide-dexamethasone—RD) schedule [67,158,159] have showed significant prolongation of PFS in phase 3 clinical trials, becoming standard salvage therapy schemes.

More recently, randomized clinical trials demonstrated a greater efficacy of triplets retaining a tolerability profile similar to that of the two-drug regimens.

The Aspire study has compared, in the MM pretreated setting, patients who underwent a combination triple therapy with carfilzomib lenalidomide and dexamethasone (carfilzomib with lenalidomide and dexamethasone KRD) to an RD schedule group. The authors reported that in the KRD cohort, there was a significant increase in responses (87% vs. 67%, *p* < 0.001) and in survival rates at two years (median PFS 26.3 months vs. 17.6 months, 95% CI: 0.57–0.83, *p* = 0.0001; OS 73% vs. 65%, 95% CI: 0.63–0.99, *p* = 0.04). KRD is associated with a slight increase in the incidence of infections and cardiac events, characterized by hypertension and seldom by heart failure and ischemic heart disease compared to KRD [156]. 

The Eloquent study showed that the combination of the anti-SLAMF7 monoclonal antibody elotuzumab with lenalidomide and dexamethasone induces a significant increase in median PFS (19.4 months vs. 14.9 months, 95% CI: 0.57–0.85; *p* < 0.001) and treatment time (TNT) (33 vs. 21 months) compared to RD in pre-treated patients. Elotuzumab-RD was very well tolerated, and infusion reactions after monoclonal antibody occur in 20% especially after the first infusion and are predominantly Grade I–II [160]. Clinical studies and the toxicity profile identify KRD as a possible choice for patients with first or second recurrence with well-controlled hypertension, without severe cardiologic comorbidities, and with adequate compliance to an intravenous treatment twice a week. Elo-RD is indicated in patients with first or second recurrence without high-risk clinical and biological features.

KRD is also indicated as a pre-transplant re-induction treatment in fit patients younger than 70 years who achieved a lasting response after autologous transplantation and who still have viable cryopreserved hematopoietic stem cells (CD34+ cells >2 × 10^6^/kg). 

Salvage autologous transplantation seems well tolerated, not very toxic, and more effective if the response of the first autologous transplant lasts longer than 18–24 months [161].

In the poor-responder/refractory patient setting, allogeneic hematopoietic stem cell transplantation (allo-HSCT) needs to be taken into account after a 4–6 KRD induction therapy. Scientific evidence indicates that heavily-pretreated patients who have failed several lines of treatment should no longer undergo allo-HSCT, as it is burdened by high transplant-related and high relapse rates. In contrast, an allo-HSCT in first recurrence for patients considered to be at high risk could maximize the advantages of the procedure, reducing toxicity and increasing the efficacy of the graft-versus-myeloma effect, although prospective studies in this patient setting are still ongoing [162]. Moreover, in RRMM, bendamustine can be used alone or in association with bortezomib in patients with preserved bone marrow reserve [163]. In more advanced stages of disease (i.e., after second relapse) pomalidomide in combination with dexamethasone represents a good treatment option [27]. Pomalidomide in combination with dexamethasone has been shown to increase PFS and OS compared to dexamethasone alone (4.1 vs. 1.9 months, 12.7 vs. 8.1 months, respectively) in RRMM patients. In terms of adverse events, a modest neutropenia and an increase rate of infections compared to the conventional arm were reported. Immunotherapy represents a novel chance for MM treatment since daratumumab [164], a specific CD38 monoclonal antibody, was added to the therapeutic armamentarium in MM. The CD38 represents a suitable antigen to target at the same time the plasma cell compartment, but also the immune-microenvironment with depletion of T and B regulatory cells and myeloid-derived suppressor cells enhancing T cell-mediated cytotoxicity [133]. The anti-CD38 monoclonal antibody daratumumab [164] has been shown to be efficient and well tolerated. In RRMM, daratumumab in monotherapy achieved at least 36% partial responses, with a PFS and OS at one year of 65 and 77%, respectively. The most important toxicity concerns infusion reactions, which are limited to the first administrations and adequately prevented by premedication with steroids and anti-H1 antihistamines. Patients with third or subsequent relapse, already exposed to proteasome inhibitors and lenalidomide, are suitable to be treated with pomalidomide and dexamethasone or to undergo salvage treatment with daratumumab.

As also mentioned above, MM is being explored in the field of the new T cell immunotherapies, as well, with the chimeric antigen receptor T cell program already targeting B-cell maturation antigen (BCMA ) [165] and with new bi-specific antibodies still in clinical trials (Figure 4A). 

### 5.2. Novel Target in Relapsed/Refractory Multiple Myeloma

The biology of RRMM patients is characterized by an acquisition of genetic lesions such as 1q amplification and deletion 17p, 1p, or 13q, usually associated with poor prognosis (Figure 4B) [95,108,176]. Moreover, oncogenes mutations such as BRAF, NRAS, and KRAS, as well as tumor suppressor genes as TP53 are enriched in the RRMM setting [108,176]. Moreover, changes in the tumoral microenvironment and the angiogenesis enhancement represent key regulators in tumor progression and refractoriness development [95]. Given the biological background, in the last few years, major improvements have been made in the treatment of this peculiar patient group. New targeted therapies are emerging in MM, such as combinations of BRAF and MEK inhibitors [177] in RAS pathway-mutated patients and BCL2 inhibitors [175,178,179]. Additionally, based on peculiar genomic features, clinical trials targeting the FGFR3, CDK, and PI3K pathways are ongoing [180] (Figure 4B). Despite encouraging pre-clinical results [181], FGFR3 inhibitors in the MM setting failed to show an effectiveness as monotherapy [174]. CDK inhibitors are the more advanced drugs in clinical trials for MM: results from a phase 1/2 study reported objective responses in 20% of patients and a stable disease maintenance in 44% [173]. These approaches are able to block the proliferative and survival advantages acquired by resistant cells during the progression of the disease and to induce deep responses also in heavily pre-treated patients [95]. Nevertheless, these new targeted approaches seem to be effective, but only in selected cases and for a limited timeframe that fit with the selection over the subclonal “underwood” that usually molecularly characterizes MM. Indeed, the association strategy will be mandatory in order to limit the overgrowth of resistant cell populations. 

## 6. Future Perspectives

An attempt to describe MM and the tumor niche genomic landscape in a patient was performed by Walker et al. with a pragmatic approach: they tried identifying the potential targetable mutations. More than 40 genetic lesions were druggable, but only three of them have already been targeted in clinical practice [182]. Although there are new therapeutic approaches for patients with high-risk MM [23,24] and the introduction of active treatments with different mechanisms of action compared to chemotherapy, therapy-sensitive patients have a very variable duration of response [93]. The MM natural history is characterized by further recurrences of diseases whose response to treatments is not durable. More effective multidrug induction regimens (e.g., PIs + IMiDs) and early high-dose therapy supported by transplant [183,184] in eligible patients did not succeed in achieving sustained response. Based on the European therapeutic approach, with short-term induction, the potential benefit of tandem ASCT compared with single ASCT is being investigated in clinical trials (NCT01208766) and could offer a better PFS and OS; in high-risk patients, an intensification through a second ASCT and a consolidation therapy with prolonged treatments can be worth exploring [185]. Moreover, maintenance therapy and immunoglobulin replacement, as infectious prophylaxis [186], can improve the clinical outcome. 

Next-generation PIs and IMiDs, as well as immunotherapy, hold promise to improve or overcome the adverse prognosis of high-risk MM and might be implemented as treatment choices in the near future [156,187,188,189,190]. Patients’ enrolment into statistically-powered prospective trials and real-life studies are of relevant importance, in order to achieve an improvement in the survival rate. The comprehensive genomic and transcriptomic characterization could lead to the identification of therapeutic targets in high-risk MM. 

## 7. Envisioned Clinical Trial and Conclusive Remarks

**Figure 5:**
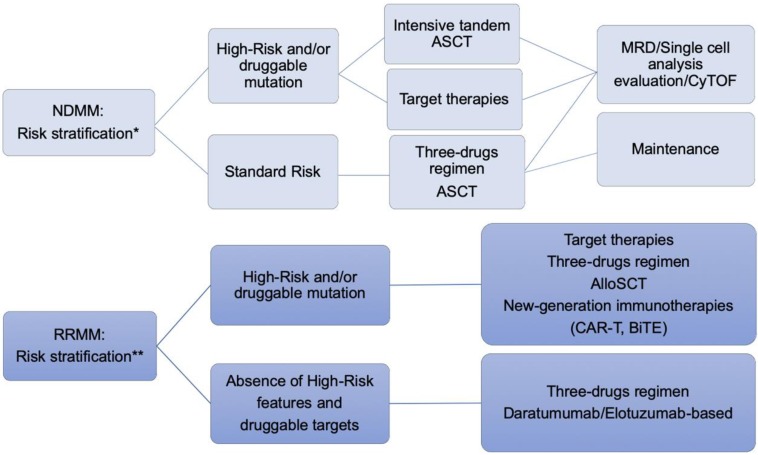
Clinical trial design proposal for a risk-driven personalized approach. NDMM: newly-diagnosed MM. RRMM: relapsed-refractory MM. ASCT: autologous stem cell transplantation. Allo-SCT: allogeneic stem cell transplantation. MRD: minimal residual disease. * FISH, NGS (genomic panels including known actionable mutation and TP53 mutation), and GEP. ** FISH, NGS (genomic panels including known actionable mutation and TP53 mutation and drug resistance-related genomic alterations), and GEP. CAR-T: Chimeric Antigen Receptor T cell; BiTE: Bispecific T cell engager antibody; tandem ASCT: double autologous stem cell transplantation. Tandem ASCT is already being investigated in experimental studies.

Our understanding of factors influencing prognosis in MM has advanced considerably. We now recognize the contribution of a range of features including patient’s baseline risk stratification, disease biology, genetic lesions, imaging findings, and depth of response. Therefore, it is reasonable to design tailored clinical trials aimed to stratify patients differentially according to disease risk. Remarkable efforts have been attempted in order to translate these unmet clinical needs to bedside-approaches [165,177,191]. As recently reported by Cavo et al. [192], TP53 mutational status and 1q amplification evaluation harbor a significant prognostic impact that can be overcome by a more aggressive therapeutic approach. In this frame of thinking, we speculated about new investigational treatments that incorporate genomic-directed stratification in both NDMM and RRMM (Figure 5). Ancillary intriguing, despite experimental evidence, techniques, such as single-cell RNA-seq analysis and mass cytometry (CyTOF), hold great promise in incorporating a comprehensive immune-microenvironment characterization into the individualized trial design and randomization [193,194,195]. 

**Figure 6:**
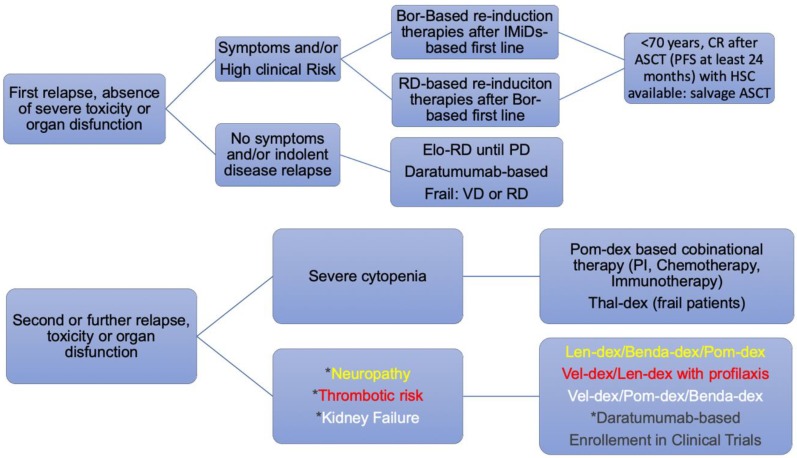
Pragmatic Integrated approach to MM patients according to the clinical risk profile. Bor: bortezomib; RD: REVLIMID^®^ (lenalidomide)-dexamethasone; Elo-RD: elotuzumab^®^-REVLIMID^®^ (lenalidomide)-dexamethasone; VD: VELCADE^®^ (bortezomib)-dexamethasone; PI: proteasome inhibitor; Thal-dex: thalidomide-dexamethasone; Len-dex: lenalidomide-dexamethasone; Benda-dex: bendamustine-dexamethasone; Pom-dex: pomalidomide-dexamethasone; Vel-dex: VELCADE^®^ (bortezomib)-dexamethasone. PI: proteasome inhibitor. PD: progressive disease; CR: complete response. PFS: progression-free survival. HSC: hematopoietic stem cells ASCT: autologous stem cell transplantation.

From a pragmatic and clinical point of view, prognostic factors can be combined to acquire a wider range of information. Early identification and a deep molecular characterization of high-risk patients at diagnosis and during the disease course can help to define an appropriate treatment strategy. Given the huge availability of newer and more effective treatments in the near future, waiting for the results of the ongoing clinical trials, we will be able to better draw a tailored therapeutic approach for the high-risk setting. Triplets including IMiDs combined with either a PI or a mAb hold promise to be effective options for high-risk MM. Cellular immunotherapies and antibody-drug conjugates or bi-specific T-cell engager antibodies are being extensively investigated in phase I–II clinical studies (Figure 6).
